# Increased Exposure of Tacrolimus by Co-administered Mycophenolate Mofetil: Population Pharmacokinetic Analysis in Healthy Volunteers

**DOI:** 10.1038/s41598-018-20071-3

**Published:** 2018-01-26

**Authors:** Jae Hyun Kim, Nayoung Han, Myeong Gyu Kim, Hwi-Yeol Yun, Sunhwa Lee, Eunjin Bae, Yon Su Kim, In-Wha Kim, Jung Mi Oh

**Affiliations:** 1 College of Pharmacy and Research Institute of Pharmaceutical Sciences, Seoul National University, Seoul, Republic of Korea; 2 College of Pharmacy, Chungnam National University, Daejeon, Republic of Korea; 3 Department of Biomedical Sciences, Seoul National University, Seoul, Republic of Korea; 4 Department of Internal Medicine, Gyeongsang National University Changwon Hospital, Changwon, Republic of Korea; 5 Kidney Research Institute, Seoul National University, Seoul, Republic of Korea; 6 Department of Medical Science, Seoul National University College of Medicine, 101 Daehak-ro, Jongno-gu, Seoul, Republic of Korea

## Abstract

The objective of the study was to investigate the pharmacokinetic drug-drug interactions between tacrolimus (TAC) and mycophenolate mofetil (MMF) in healthy Korean male volunteers. Seventeen volunteers participated in a three-period, single-dose, and fixed sequence study. They sequentially received MMF, TAC, and the combination. Concentrations of TAC, mycophenolic acid (MPA), and its metabolites MPA 7-O-glucuronide and MPA acyl glucuronide were measured. The variants of *CYP3A4*, *CYP3A5*, *SLCO1B1*, *SLCO1B3*, *ABCC*2, *UGT1A*9, and *UGT2B7* were genotyped. Drug interaction was evaluated with a non-compartmental analysis and population pharmacokinetic modelling to quantify the interaction effect. A total of 1,082 concentrations of those analytes were analysed. AUC_0-inf_ of TAC increased by 22.1% (322.4 ± 174.1 to 393.6 ± 121.7 ng·h/mL; *P* < 0.05) when co-administered with MMF, whereas the pharmacokinetic parameters of MPA and its metabolites were not changed by TAC. Apparent clearance (CL/*F*) of TAC was 17.8 L/h [relative standard error (RSE) 11%] or 13.8 L/h (RSE 11%) without or with MMF, respectively. Interaction was explained by the exponential model. The *CYP3A*5 genotype was the only significant covariate. The population estimate of CL/*F* of TAC was 1.48-fold (RSE 16%) in *CYP3A5* expressers when compared to nonexpressers. CL*/F* of TAC was decreased when co-administered with MMF in these subjects.

## Introduction

Tacrolimus (TAC) and mycophenolate mofetil (MMF) are both prescribed for prevention of rejection after solid organ transplantation^1^. Previous clinical trials have reported that the combination of TAC and MMF demonstrates significant improvement in transplant outcomes due to a synergistic effect based on different mechanisms of action^2–4^. The use of a triple immunosuppression regimen consisting of calcineurin inhibitors, antiproliferative agents, and steroids is recommended after transplantation^5^. The combination of TAC and MMF is used most frequently for prevention of graft rejection after transplantation^6^. However, because the two drugs have narrow therapeutic ranges and high individual variability in their pharmacokinetics, it is important to determine the accurate dose considering patient characteristics^7,8^. Frequent discontinuation and resumption of immunosuppressants due to adverse events or infection may further complicate precision of dose adjustment because of change in the effect of drug interaction^9^.

The results of several recent studies suggested that drug interactions may occur when TAC is combined with MMF. Pharmacokinetics may be altered by inhibition of drug metabolism or induction of clearance^10,11^, by mediating transporters and enzymes^12^. Meanwhile, another study revealed no significant changes in pharmacokinetic parameters of TAC and MMF^13^. The reason for the ongoing and decades-long controversy concerning the drug-drug interaction between TAC and MMF seems to be due to a few drug metabolizing enzymes or transporters commonly involved in the metabolic pathway of both drugs^14,15^. The cytochrome P450 3A5 (*CYP3A5*) genotype markedly influences the pharmacokinetics of TAC, while uridine glucuronosyl transferase (UGT) and solute carrier organic anion transporter (SLCO) genotypes have been related with significantly increased dose-adjusted mycophenolic acid (MPA) trough levels^16–18^. There is a lack of evidence for drug interaction in terms of pharmacokinetic parameters. In particular, the possible drug interaction between TAC and MMF and factors related to the effect of interaction have never been investigated in human subjects.

Therefore, a new study is needed to investigate the pharmacokinetic interaction by comparing pharmacokinetic parameters between monotherapy and combination administration. To find the factors that contribute to pharmacokinetic interaction between TAC and MMF, population pharmacokinetic modelling is useful. Evaluation of the influence of drug interactions on the pharmacokinetic changes enables fine dose adjustment in patients treated with TAC and MMF.

The purpose of this study was to investigate the interaction between TAC and MMF in healthy volunteers using linear and nonlinear models. We also planned to assess the magnitude of pharmacokinetic interaction by clinical covariates or genotypes.

## Results

### Demographics

Eighteen healthy volunteers were enrolled in this clinical trial. One participant dropped out due to an acute kidney injury sustained after the single dose of MMF in the first period. Baseline demographic characteristics of the remaining 17 volunteers are summarised in Table 1. The median age of the subjects was 25 years (range 20 to 42). Genotype and allele frequencies are presented in Supplementary Table S2. All genotype frequencies were within Hardy-Weinberg equilibrium (*P* > 0.05). Four participants (23.5%) were *CYP3A5* expressers (CYP3A5*1/*1 or CYP3A5*1/*3).

**Table 1:** Baseline characteristics of enrolled healthy volunteers (*n* = 17).

**Characteristics**	**Median**	**Range (min-max)**
Age (yr)	25	20–42
Weight (kg)	69.7	57.4–88.3
Height (cm)	173.4	167.7–192.8
Hemoglobin (g/dL)	15	13.6–16.2
Hematocrit (%)	44.6	41.0–47.4
ANC (/μL)	3,022	1,553–5,858
Serum creatinine (mg/dL)	0.86	0.79–1.20
GFR (mL/min/1.73 m^2^)	104.8	74.1–122.3
Albumin (g/dL)	4.6	4.2–4.9
Total bilirubin (mg/dL)	0.8	0.5–1.2
*CYP3A5* expresser^a^	4	23.5%

^a^*CYP3A5* expresser is presented as number and proportion.

ANC, absolute neutrophil count; *CYP3A5* expresser, *CYP3A5* *1/*1 or *CYP3A5* *1/*3; GFR, glomerular filtration rate.

### Non-compartmental analysis

A total of 1,082 concentrations were measured for four compounds, with an average of 63 concentration data per subject. Area under the blood concentration-time curve (AUC) from time 0 to infinity (AUC_0-inf_) of TAC was increased significantly in combination with TAC and MMF, as compared with TAC alone (combination *vs*. monotherapy, 393.6 ± 121.7 *vs*. 322.4 ± 174.1 ng·h/mL, *P* < 0.05) (Table 2). Maximum concentration (C_max_) was increased from 33.5 ± 10.6 ng/mL to 35.0 ± 12.5 ng/mL in combination treatment, but the difference was not statistically significant (*P* = 0.58). No significant changes in pharmacokinetic parameters of MPA and its metabolites MPA 7-O-glucuronide (MPAG) and MPA acyl glucuronide (AcMPAG) were observed when comparing the administration of MMF alone or with TAC (Supplementary Table S3). Based on the non-compartmental analysis results, the apparent clearance (CL/*F*) of TAC was decreased significantly by co-administration with MMF (combination *vs*. monotherapy, 14.2 ± 5.8 *vs*. 20.1 ± 10.7 L/h, *P* < 0.05).

**Table 2:** Pharmacokinetic parameters of tacrolimus estimated by the non-compartmental analysis after administration of TAC alone or in combination with MMF.

**Parameter**	**TAC (mean ± SD)**	**TAC + MMF (mean ± SD)**	***P*** **value**
C_max_ (ng/mL)	33.5 ± 10.6	35.0 ± 12.5	0.5825
*t*_max_ (h)	1 (1–2)	2 (1–4)	—
AUC_0–12_ (ng·h/mL)	139.3 ± 61.7	167.3 ± 54.9	<0.05
AUC_0–24_ (ng·h/mL)	180.2 ± 85.0	221.8 ± 71.0	<0.05
AUC_0–48_ (ng·h/mL)	235.1 ± 117.4	292.0 ± 91.5	<0.05
AUC_0–72_ (ng·h/mL)	267.9 ± 137.2	333.0 ± 103.9	<0.05
AUC_0-inf_ (ng·h/mL)	322.4 ± 174.1	393.6 ± 121.7	<0.05
CL*/F* (L/h)	20.1 ± 10.7	14.2 ± 5.8	<0.05

TAC, tacrolimus; MMF, mycophenolate mofetil; SD, standard deviation; C_max_, maximum concentration; *t*_max_, time of maximum concentration; AUC, area under the blood concentration-time curve from time 0 to infinity or pre-specified time points; *CL/F*, apparent clearance.

CL*/F* was calculated from dose and AUC_0-inf_; *t*_max_ is presented as median (min-max); *P* value was obtained by paired *t*-test or signed rank test.

### Individual population pharmacokinetic model development

**Figure 1:**
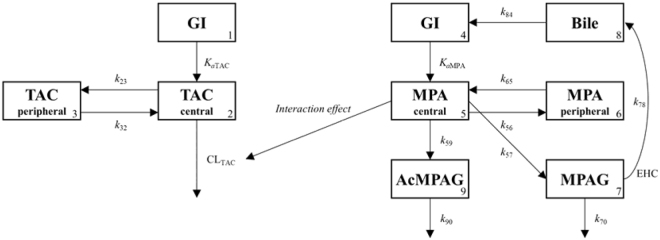
Schematic presentation of an integrated population pharmacokinetic model. Compartments: gastrointestinal tract (GI, 1, 4), central compartment for tacrolimus (2); peripheral compartment for tacrolimus (3), central compartment for mycophenolic acid (5), peripheral compartment for mycophenolic acid (6), compartment for mycophenolic acid 7-O-glucuronide (7), compartment for gallbladder (8), compartment for mycophenolic acid acyl glucuronide (9). TAC, tacrolimus; *K*_a_, absorption rate constant; *k*_23_, *k*_32_, *k*_56_, and *k*_65_, intercompartment rate constants; *CL*, clearance; MPA, mycophenolic acid; MPAG, MPA 7-O-glucuronide; AcMPAG, MPA acyl glucuronide; *k*_57_ and *k*_59_, metabolized rate constants for mycophenolic acid; EHC, enterohepatic circulation; *k*_78_, biliary recirculation of MPAG into GI; *k*_70_ and *k*_90_, eliminated rate constants; *k*_84_, gallbladder emptying rate constant; Meal times were used to trigger timing of gallbladder emptying.

Among the drug concentration data, 5 of TAC, 28 of MPA, 1 of MPAG, and 92 of AcMPAG concentrations were detectable but were below limit of quantification (BLQ). The structure of individual and integrated population pharmacokinetic models is presented in Fig. 1.

A two-compartment, first-order absorption with lag time, and first-order elimination model best explained the pharmacokinetics of TAC alone. The estimated population mean value, relative standard error (RSE), and interindividual variability (IIV) are presented in Table 3. Population mean value of CL*/F*, *V*_2_*/F* and absorption rate constant (*K*_a_) was 17.8 L/h, 108 L, and 3.75 h^−1^, respectively. The proportional error model was used for residual error. Through covariate searching, *CYP3A*5 was the only covariate that significantly improved the pharmacokinetic model of TAC (change of objective function value, ∆OFV = 6.761). In *CYP3A5* expressers, CL*/F* of TAC was increased by 1.26-fold compared to *CYP3A5* nonexpressers.

**Table 3:** Population pharmacokinetic parameter estimates of models for TAC and MMF.

**Parameter**	**Independent model**		**Integrated model**	
	**Population mean value (%RSE)**	**IIV CV% (%RSE)**	**Population mean value (%RSE)**	**IIV CV% (%RSE)**
***Tacrolimus***				
*CL/F*_TAC_ (L/h)	17.8 (11%)	50.9% (14%)	13.8 (11%)	26.4% (19%)
*V*_*2*_*/F* (L)	108 (12%)	44.4% (13%)	93 (9%)	30.8% (13%)
*K*_*a*TAC_ (h^−1^)	3.75 (60%)	160% (36%)	1.78 (43%)	93% (18%)
*k*_23_ (h^−1^)	0.326 (4%)	—	0.313 (6%)	—
*k*_32_ (h^−1^)	0.069 (5%)	—	0.0719 (6%)	—
Lag time (h)	0.627 (26%)	—	0.59 (30%)	—
*CYP3A5 on CL/F*	1.26 (7%)	—	1.48 (16%)	—
*σ*_prop TAC_	0.131 (13%)	—	0.131 (12%)	—
***Mycophenolic acid***				
*CL/F*_MPA_ (L/h)	16.1 (7%)	25.9% (35%)	16.3 (7%)	18.7% (32%)
*V*_*5*_*/F* (L)	16.8 (12%)	40.9% (28%)	19.7 (11%)	18.2% (27%)
*K*_*a*MPA_ (h^−1^)	2.06 (14%)	69.7% (17%)	2.29 (9%)	56.6% (28%)
*k*_56_ (h^−1^)	1.33 (6%)	—	1.12 (10%)	—
*k*_65_ (h^−1^)	0.109 (9%)	—	0.131 (7%)	—
*k*_70_ (h^−1^)	0.256 (10%)	—	0.251 (13%)	—
*V*_*7*_*/F* (L)	5.13 (7%)	—	5.83 (7%)	—
f_MPA_	0.85 fix	—	0.85 fix	—
EHC (%)	0.427 (10%)	30.1% (22%)	0.367 (15%)	35.5% (18%)
*k*_84_ (h^−1^)	263 (540%)	—	18.4 (160%)	—
MTIME1	7.99 (0%)	—	7.96 (1%)	—
MTIME2	1 fix	—	1 fix	—
*V*_*9*_*/F* (L)	23 fix	—	23 fix	—
*k*_90_ (h^−1^)	2.15 fix	—	2.15 fix	—
*σ*_prop MPA_	0.516 (9%)	—	0.524 (7%)	—
*σ*_add MPAG_	0.186 (44%)	—	0.104 (31%)	—
*σ*_prop MPAG_	0.172 (8%)	—	0.237 (12%)	—
*σ*_prop AcMPAG_	0.654 (31%)	—	0.651 (22%)	—
Interaction	—	—	0.0294 (154%)	—

IIV, interindividual variability; CV, coefficient of variation; RSE, relative standard error; F, fraction of the dose absorbed; *CL/F*, apparent clearance; TAC, tacrolimus; *V/F*, apparent volume of distribution; *K*_a_, first-order absorption rate constant; *k*_23_, *k*_32_, *k*_56_, and *k*_65_, intercompartment rate constants; MPA, mycophenolic acid; *k*_70_ and *k*_90_, eliminated rate constants; *CYP3A5*, *CYP3A5* expressers (CYP3A5*1/*1 or CYP3A5*1/*3); f_MPA_, fraction of MPA which metabolized to MPAG; EHC, enterohepatic circulation; *k*_84_, gallbladder emptying rate constant; MTIME1, meal time; MTIME2, Gallbladder emptying duration; MPAG, MPA 7-O-glucuronide; AcMPAG, MPA acyl glucuronide; *σ*_prop,_ proportional residual error; *σ*_add,_ additive residual error.

Pharmacokinetic data of MPA, MPAG, and AcMPAG were modeled sequentially. The MPA concentration-time profile was best described by a two-compartment model with first-order absorption. Concentration data of MPAG was then combined with the MPA structure model, concerning the enterohepatic circulation (EHC) process estimated as: (1) fraction of drug following this route and (2) gallbladder emptying time. The model with the gallbladder compartment was better than that without the gallbladder compartment with respect to physiological plausibility and model stability. The gallbladder emptying process was described by parameters including meal time (MTIME1), gallbladder emptying duration (MTIME2), rate constant (*k*_84_), and fraction of drug undergoing EHC (equation (1)).

1 $$EHC( \% )=\frac{{k}_{78}}{{k}_{70}+{k}_{78}}\times 100$$

To reflect the actual meal time, EHC models with two or more meal times were tested. They failed to converge. Gallbladder emptying time was estimated to be 1 h and was fixed based on previous reported pharmacokinetic model^19^. To estimate *k*_84_ as transfer rate constant from gallbladder to the absorption compartment, the fraction of MPA that metabolised to MPAG was fixed to 85%^20–22^. Final estimates of parameters explaining the process of EHC were 7.99 h (RSE 0%, MTIME1), 263 h^−1^ (RSE 540%, *k*_84_), and 42.7% (RSE 10%, EHC).

We assumed that the elimination of MPA by kidney appears as the excretion of AcMPAG. The estimated elimination rate constant and *V*_*9*_/*F* of AcMPAG using the Laplacian with interaction (LAPL + I) method was 2.15 h^−1^ and 23 L, respectively, and finally it was fixed. No covariates were selected in the stepwise covariate modelling of MMF.

### Interaction model development

Developed individual TAC and MPA models were integrated to assess the effect of the interaction between the two. The concentration of MPA had an inverse exponential relationship with the CL*/F* of TAC. The estimated slope value was 0.0294 (RSE 154%). After interaction effect was accounted for, CL/*F* of TAC was decreased from 17.8 L/h (RSE 11%) in a single dose model to 13.8 L/h (RSE 11%) in the combination model. In the final population pharmacokinetic model of TAC, the *CYP3A5* genotype was identified as a significant covariate in the *CL/F* as follows (equation (2)):

2 $$CL/F(L/h)=13.8\times \frac{1}{{e}^{0.0294\bullet {C}_{MPA}}}\times {1.48}^{CYP3A5}$$

where the value of *CYP3A5* is 1 in *CYP3A5* expressers, and 0 in otherwise, and C_MPA_ is the concentrations of MPA.

### Model evaluation

**Figure 2:**
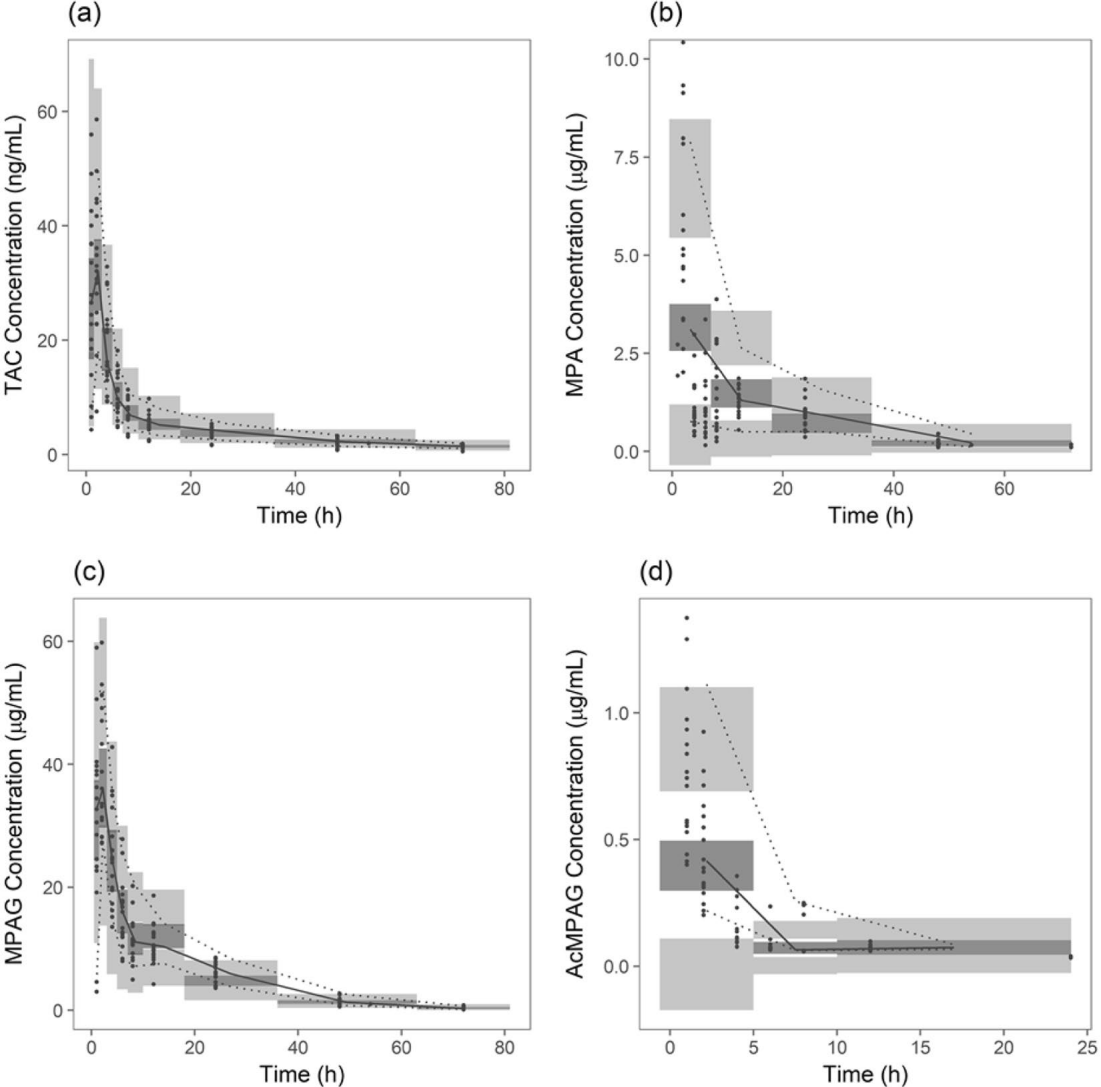
Visual predictive check of integrated population pharmacokinetic models. (**a**) TAC; (**b**) MPA; (**c**) MPAG; (**d**) AcMPAG. Closed circles represent observed concentrations. Solid line represents median of observed concentration and dotted line represents 5^th^ and 95^th^ percentile of observed concentration. Light grey area represents 95% confidence interval of 5^th^ and 95^th^ percentile of predicted concentration and dark grey area, 95% confidence interval of median of predicted concentration. TAC, tacrolimus; MPA, mycophenolic acid; MPAG, MPA 7-O-glucuronide; AcMPAG, MPA acyl glucuronide.

The diagnostic plots of the final models indicated acceptable goodness-of-fit (Supplementary Figures S1–S4). The developed model well predicted the population and individual value of measured concentration. Plot of conditional weighted residuals (CWRES) versus time or population prediction (PRED) did not show any trend, with most of absolute CWRES values were below 3. Eta shrinkage of the integrated model were all below 20%, except for 27% for *V*_*5*_*/F*. The visual predictive check (VPC) plot of each compound showed that 95% confidence interval includes the observed concentrations, thereby confirming the predictability of developed model (Fig. 2).

## Discussion

In this study, we evaluated the pharmacokinetic interactions between TAC and MMF in healthy volunteers using the non-linear mixed effect model. This is the first study to investigate the magnitude and characteristics of the interaction with non-compartmental analysis and to quantify the effect of interaction with the population pharmacokinetic model. Beyond the simple evaluation based on comparison of AUC, we estimated the interaction effect and identified clinical factors associated with the interaction. As the concentration of MPA increased by 5 μg/mL CL*/F* and the corresponding required dose of TAC decreased by 13.7%, regardless of *CYP3A5* genotypes, according to the integrated population pharmacokinetic model. With respect to *CYP3A5* genotype, CL*/F* of TAC was 1.48-fold in *CYP3A5* expressers when compared to nonexpressers.

The final population model of TAC characterised its pharmacokinetics with the two-compartment and first-order absorption model. *CYP3A5* genotype was the only covariate included in this model, which was repeatedly identified as a significant covariate in previous population pharmacokinetic models of TAC^16,23–25^. Estimated values of structural parameters were also comparable to previous reported models^24,25^. All subjects were healthy male adults. Variation of their baseline demographics was small, so the effects of other covariates were not significant.

The final model of MMF consisted of MPA and its metabolites including MPAG and AcMPAG. Population pharmacokinetic estimates of metabolites were similar when compared with those of previous developed models^19,20^. To develop a more physiologic model, additional gallbladder compartment was incorporated to characterise EHC of MPA. Gallbladder emptying was designed to occur at once with fixed duration of 1 h. We tried to set multiple emptying times to mimic the actual meal time in the clinical trial, but the model with two or more gallbladder emptying times was unstable. Scarce sampling time point around actual meal time might be the cause of the estimation failure. Considering that the concentration-time profile of MPA shows a secondary peak at 6–12 h after administration, a model with a gallbladder emptying time at 7.99 h is also reasonable^26^. The concentration of AcMPAG had a negligible effect on the concentrations of MPA or on the interaction between MMF and TAC, thus typical population pharmacokinetic parameters of AcMPAG were fixed after initial estimation with the LAPL + I estimation method.

In the interaction model, the interaction between TAC and MMF was explained by an inverse exponential relationship. Decrease in CL*/F* of TAC might be due to inhibition of metabolic enzymes by co-administered MMF. In human liver microsome study by Picard *et al*., metabolism of TAC was inhibited in the presence of MPA^10^. Braun *et al*. also suggested that interaction might occur through unknown effect of CYP3A, multidrug resistance-associated protein 2 (MRP2) or UGT based on the results of clinical study^12^.

In other studies that evaluated interaction by the population pharmacokinetic model, the effect of interaction was usually modelled as a binary covariate with an on-off status depending on the administration of interacting drug^27^. Another study introduced the IIV term to evaluate the interaction effect as IIV in structural parameter estimates^28^. Dealing with the interaction effect as a simple covariate accompanies loss of information and thus has disadvantages in characterising the course of interaction and estimating the precise effect. Availability of detailed information regarding the co-administered drug such as dosing regimen, administration time, and concentrations often limits the methods of evaluating interaction effects in the population pharmacokinetic model. In our research, the concentration of TAC as well as MMF was measured, thus the dynamic course of interaction was observable.

The *CYP3A5* genotype was identified as the significant covariate in the independent TAC model and in the integrated model, which accounted for the effect of interaction with MPA. The role of *CYP3A5* enzyme on pharmacokinetics of TAC has extensively been investigated in previous studies^29^. Some recent studies evaluated interaction of TAC in a stratified population according to metabolic enzyme polymorphisms^30–32^. In the study of Zuo *et al*., co-administration with amlodipine resulted in a significant decrease of TAC clearance only in *CYP3A5* expressers^32^. Genotype dependent interaction effect was also observed and *CYP3A5* genotype was a more influential covariate compared to co-administration of MMF for the clearance of TAC in our study.

Evaluation of interaction in healthy volunteers rather than in target patient population might limit the generalisability of the study result. According to relevant international guidelines, however, it is recommended that interaction be evaluated in healthy volunteers^33,34^. Moreover, it would be unethical to discontinue either one of the two immunosuppressants to study drug-drug interaction in transplant recipients. Another limitation is that the study drug was administered once rather than on multiple occasions. Although the drug interaction is generally evaluated at steady state concentrations of perpetrator drug^34^, it was difficult to discern the “perpetrator and victim” relationship solely based on previous *in vitro* or *in vivo* studies. Therefore, we planned to conduct a study with single dose to allow qualitative screening at least. Another study, which evaluated interaction between TAC and sirolimus, also adopted the single dose study design^35^.

In conclusion, this study identified the interaction between TAC and MMF with the integrated population pharmacokinetic model. Concentrations of TAC can be increased with co-administration with MMF. This effect is augmented in *CYP3A5* expressers. Considering that recent clinical trials evaluate various combinations of TAC and MMF^36–38^, characterising the pharmacokinetic interaction between these two drugs is important. A developed population pharmacokinetic model can be used to predict the concentration of TAC and MMF in various dosage combinations while considering the effect of interaction at the same time. However, further research is necessary to confirm whether the effect of drug-drug interaction persists in the target patient population.

## Methods

### Study design and population

The study was a three-period, fixed sequence, open-label, and single-dose clinical trial conducted in 2015 and 2016. This study involved healthy volunteers who met the following conditions: male adults ranging in age from 19 to 45 years, less than 20% differential between actual body weight and ideal body weight, and no known history of previous disease. Volunteers were also required to be normal on physical exam and laboratory tests (electrolytes, complete blood counts, renal and hepatic function tests, and electrocardiogram). The estimated glomerular filtration rate was calculated by the Modification of Diet in Renal Disease (MDRD) equation^39^. Volunteers were excluded if they had taken any other drugs or herbal medicines within the one month period immediately antedating the study, as well as those who had been heavy-drinkers during the month preceding the study. Subjects who had smoked within one year of the commencement date of the study were also disqualified. All subjects in this study were of Korean ethnicity.

### Ethical issue

The study was conducted in accordance with the Declaration of Helsinki, the International Conference on Harmonization Guidelines for Good Clinical Practice^40^. This study was approved by the institutional review board (IRB No. C-1506-155-686) of Seoul National University Hospital (Seoul, Korea) and all subjects were given written informed consent before undergoing this study procedure (Clinicaltrials.gov identifier NCT02743247; Date of registration 18/12/2015).

### Drug administration

In the first period, subjects received 1,000 mg MMF (Cellcept^®^; Roche Korea, Seoul, Korea) alone. After a week, subjects received 5 mg TAC (Prograf ^®^; Astellas Pharma Korea Inc., Seoul, Korea) alone, and then a combination of 1,000 mg MMF and 5 mg TAC with a one-week washout period. Study drugs were administered with 240 mL water in the fasting state. All subjects were maintained in a strictly upright position until 2 h after drug administration. Water and food were allowed 2 and 4 h after drug administration. Subjects were also required to abstain from alcohol, grapefruit, and any other medicinal products during the study period.

### Bioanalytical methods

Blood samples were drawn predose and postdose 1, 2, 4, 6, 8, 12, 24, 48, 72, and 168 h after drug administration. All samples were collected in ethylenediaminetetraacetic acid dipotassium salts containing tubes. Blood samples for MPA and its metabolites were centrifuged at 3,000 rpm and 4 °C for 15 min to obtain plasma. Plasma was acidified with phosphoric acid (850 g/L; Sigma-Aldrich, St. Louis, MO) to pH 2.5 to stabilise the metabolites of MPA^41^. Whole blood and plasma samples were stored at −70 °C until analysis.

TAC concentration was measured with the validated liquid chromatography/tandem mass spectrometry (LC-MS/MS) in whole blood samples. The LC system (Shimadzu Corp. Tokyo, Japan) coupled with an AB SCIEX QTRAP^®^ 5500 triple-quadrupole mass-spectrometric system (AB Sciex, Foster City, CA). The instrument was set to collect data in multiple reaction monitoring mode using electrospray ionisation positive mode. Rapamycin served as the internal standard. TAC and the internal standard were separated by Capcell PAK MGIII (3.0 × 50 mm, 3 μm; Shiseido, Tokyo, Japan) column. The mobile phase consisted of 5 mM ammonium acetate (A) and methanol (B) with gradient elution; A:B was as follows: 0 min, 65:35; 2 min, 0:100; 2.5 min, 65:35; and maintained for 3.5 min. Flow rate was 0.35 mL/min and 5 μL of treated sample was injected into the LC-MS/MS system. The standard curves for TAC were linear over the range of 0.5–100 ng/mL. TAC concentration was linear and accurate in the range of analysis with coefficient of variation (CV) less than 6%.

Concentrations of MPA, MPAG, and AcMPAG were analysed with the validated LC-MS/MS method in acidified plasma samples. MPA-d3, MPA-d3 β-D-glucuronide, or MPA-d3 acyl-β-D-glucuronide was used respectively, as an internal standard for each analyte. Analytes were separated by Cadenza CD-C18 column (3.0 × 150 mm, 3 µm; Imtakt, Kyoto, Japan). The mobile phase consisted of 0.1% formic acid in 5 mM ammonium acetate (A) and acetonitrile (B) with gradient elution; A:B was as follows: 0 min, 90:10; 4 min, 60:40; 8 min, 40:60; 9 min, 5:95; 10 min, 90:10; and maintained for 2 min. Flow rate was 0.35 mL/min and 5 μL of treated sample was injected into the LC/MS/MS system. The standard curves for MPA, MPAG, and AcMPAG were linear over the range of 0.1–100, 0.1–100, and 0.2–20 μg/mL, respectively. Concentration was linear and accurate in the range of analysis with CV less than 5%.

### Genotyping and data collection

Genomic DNA was extracted from whole blood samples using the QIAamp DNA blood kit (Qiagen, Valencia, CA) according to the manufacturer’s protocol. Genotypes of CYP3A4*1 G (rs2242480), CYP3A5*3 (rs776746), SLCO1B1*1B (rs2306283), SLCO1B1*5 (rs4149056), *SLCO1B3* 334 T > G (rs4149117), *SLCO1B3* 699 G > A (rs7311358), *ABCC2* –24 C > T (rs717620), *ABCC2* 1249 G > A (rs2273697), *ABCC2* 3972 C > T (rs3740066), and *UGT2B*7 802 C > T (rs7439366) were determined by ABI PRISM SNaPshot Multiplex kit (Applied Biosystems, Foster City, CA) according to the manufacturer’s protocol. *UGT1A*9*1b (rs3832043) genotype was determined by direct sequencing. Primer sequences and experimental conditions are provided in Supplementary Table S1. Deviation of the observed genotype distribution from the Hardy-Weinberg equilibrium was tested using the chi-square test. Clinical variables including age, body weight, height, serum creatinine, glomerular filtration rate, haemoglobin, haematocrit, albumin, total bilirubin, and absolute neutrophil count were collected at the time of drug administration.

### Non-compartmental analysis

Non-compartmental analysis was used to determine individual pharmacokinetic parameters in all periods. Pharmacokinetic parameters including the C_max_, time to C_max_ (*t*_max_), oral clearance and volume of distribution expressed as a function of bioavailability (CL*/F* and *V/F*) and AUC from time 0 to infinity or until 12, 24, 48, and 72 h after administration were estimated. The pharmacokinetic parameters were estimated with WinNonLin version 6.4 (Pharsight, Mountain View, CA). All pharmacokinetic parameters of TAC, MPA, and its metabolites estimated from a single dose period were compared with those from the subsequent combination period. Statistical significance was tested with paired *t*-test or signed rank test based on the result of Shapiro-Wilk’s normality test. Statistical analyses were performed with R software version 3.2.2 (www.r-project.org).

### Population pharmacokinetic models development

Individual population pharmacokinetic model of TAC and MMF was independently developed before the estimation of an interaction effect in the integrated model. The user defined subroutine ADVAN6 with differential equations was used to estimate the typical population parameters. In each model, one or two compartment models were tested to explain drug pharmacokinetics. To characterise the absorption process of each drug, various absorption models including first order absorption with or without lag time or a transit compartment model were tested. In the case of the MMF model, various types of models including transfer rate constant, gallbladder compartment, and model event time parameter were tested to consider EHC of MPAG, which is excreted into the bile and subsequently reabsorbed as MPA. IIV related to pharmacokinetic parameters was assumed to be log-normally distributed and modelled as an exponential relationship. Additive, proportional, and combined additive and proportional error models were tested to specify residual variability of model.

LAPL + I or first-order conditional estimation with interaction (FOCE + I) method was used depending on the observed fraction of BLQ concentrations^42^. For each compound, if less than 10% of total observations were in the range of BLQ, FOCE + I estimation method was used after omitting those observations^43^. On the other hand, LAPL + I estimation method was used and BLQ observations were replaced with individual predicted values. All population pharmacokinetic modelling was performed using NONMEM version v.7.3.0 (ICON Development Solutions, Hanover, MD).

Clinical covariates, as well as genotypes, were tested on CL*/F* and *V/F* using linear, power, and exponential functions. Continuous covariates were centred on their median values and categorical covariates were coded as binary variables. Relationship between parameters and covariates was explored with a stepwise covariate model (SCM) procedure in Perl-speaks-NONMEM (PsN) version 4.4.8^44,45^. Significant covariate was selected by comparing OFV of two nested models. Statistical significance of difference in OFV was evaluated with a likelihood ratio test and test statistics was assumed to follow chi-square distribution. In a forward inclusion and backward elimination steps, the ΔOFV of more than 3.84 (*P* < 0.05) and 6.63 (*P* < 0.01) were regarded as statistically significant, respectively.

### Interaction model development

The TAC and MMF model was combined to form the full integrated model. To estimate the effect of interaction between TAC and MPA, linear (equation (3)), exponential (equation (4)) or E_max_ (equation (5)) model was tested. Backward elimination process was done to exclude non-significant covariates after combining the two models.

3 $$Interaction=Slope\bullet {C}_{drug}$$

4 $$Interaction=\frac{1}{{e}^{Slope\bullet {C}_{drug}}}$$

5 $$Interaction=\frac{{E}_{{\rm{\max }}}\bullet {C}_{drug}}{E{C}_{50}+{C}_{drug}}$$

### Model evaluation

Determination of model improvement was guided not only by OFV, but with scientific plausibility of estimated parameter value, relative standard error, shrinkage and goodness-of-fit plots including observed (DV) versus individual prediction (IPRED), DV versus PRED, CWRES versus time and CWRES versus PRED were used for diagnostic purposes. Prediction-corrected VPC based on 1,000 simulations was used to test the internal validity of all developed models^46^. The goodness-of-fit was assessed by graphical diagnostics using Xpose 4 in R software version 3.2.2^47^.

## Electronic supplementary material

Supplementary Information
