# Supplementary material for: Increased Exposure of Tacrolimus by Co-administered Mycophenolate Mofetil: Population Pharmacokinetic Analysis in Healthy Volunteers
Source: Sci Rep. 2018 Jan 26;8:1687. doi: 10.1038/s41598-018-20071-3 (PMC5786104; doi:10.1038/s41598-018-20071-3)
Supplement: Supplementary file 1 — Supplementary Information [file 41598_2018_20071_MOESM1_ESM.pdf]

**Increased Exposure of Tacrolimus by Co-administered Mycophenolate Mofetil: Population Pharmacokinetic Analysis in Healthy Volunteers**

Jae Hyun Kim[1], Nayoung Han[1], Myeong Gyu Kim[1], Hwi-Yeol Yun[2], Sunhwa Lee[3], Eunjin Bae[4], Yon Su Kim[5,6], In-Wha Kim[1] and Jung Mi Oh[1*]

[1]College of Pharmacy and Research Institute of Pharmaceutical Sciences, Seoul National University, Seoul, Republic of Korea

[2]College of Pharmacy, Chungnam National University, Daejeon, Republic of Korea

[3]Department of Biomedical Sciences, Seoul National University, Seoul, Republic of Korea

[4]Department of Internal Medicine, Gyeongsang National University Changwon Hospital, Changwon, Republic of Korea

[5]Kidney Research Institute, Seoul National University, Seoul, Republic of Korea

[6]Department of Medical Science, Seoul National University College of Medicine, 101 Daehak-ro, Jongno-gu, Seoul, Republic of Korea

***Corresponding Author**

Jung Mi Oh, College of Pharmacy, Research Institute of Pharmaceutical Sciences, Seoul National University, 1 Gwanakro, Gwanak-gu, Seoul 08826, Republic of Korea. Tel.: +82 2 880 7997; fax: +82 2 882 9560; email address: jmoh@snu.ac.kr

**Supplementary Table S1** Primer sequence and experimental condition for genotyping

| Gene | rs number | Primer | Sequence | Tm |
|---|---|---|---|---|
| *CYP3A4* | rs2242480 | Forward | CCAGCAGAAACTGCAGG | 60 |
| | | Reverse | GAGTCAGTGAAAGAATCAGTGATT | |
| | | Genotyping | TACCCAATAAGGTGAGTGGATG | |
| *CYP3A5* | rs776746 | Forward | TTATGGAGAGTGGCATAGGA | 60 |
| | | Reverse | GCTGATTAAACTTCACTAGCC | |
| | | Genotyping | CTCTTTAAAGAGMTCTTTTGTCTTTCA | |
| *SLCO1B1* | rs2306283 | Forward | TGTTCTTACAGTTACAGGTATTC | 55 |
| | | Reverse | TCCAGTTCAGAtggacaaa | |
| | | Genotyping | atgttgaaKtttctgatgaat | |
| *SLCO1B1* | rs4149056 | Forward | tgtcaaagtttgcaaagtg | 60 |
| | | Reverse | GGACCAATCATTGCTATTG | |
| | | Genotyping | TCTGGGTCATACATGTGGATATRTG | |
| *SLCO1B3* | rs4149117 | Forward | ttgagggaaggtacaatgtc | 55 |
| | | Reverse | ggtgaagttgtgaagcctta | |
| | | Genotyping | TGGGAAMTGGAAGTATTTTGACA | |
| *SLCO1B3* | rs7311358 | Forward | CTGGATCTACCCTTGAAAT | 55 |
| | | Reverse | GATTATTAATGGATTTATTTCCTAC | |
| | | Genotyping | GATCTACATATCCAATATCCACGTA | |
| *ABCC2* | rs717620 | Forward | TGTACTTTGGGAACTGGTG | 55 |
| | | Reverse | TCTGGTTCTTGTTGGTGAC | |
| | | Genotyping | catgattcctggactgcgtctggaaY | |
| *ABCC2* | rs2273697 | Forward | tggaggaaggtgggaataa | 55 |
| | | Reverse | GGTCCCAACTCTCTCCATA | |
| | | Genotyping | CCAACTTGGCCAGGAAGGAGTACACC | |
| *ABCC2* | rs3740066 | Forward | CCCTGGGTGACTGATAAGA | 55 |
| | | Reverse | TCCCTCTGATACTGTGTCC | |
| | | Genotyping | TCCTCAGAGGGATCACTTGTGACAT | |
| *UGT1A9* | rs3832043 | Forward | AGGCGAGCCCCAATTTAG | 60 |
| | | Reverse | CAAAGCCACAGGTCAGCA | |
| *UGT2B7* | rs7439366 | Forward | TTTCAAAGCACAGATATTTGCCT | 60 |
| | | Reverse | gtgttttgtgctaatccctttgt | |
| | | Genotyping | CGAAACTCCTGGAATTTTCAGTTTCCW | |

Tm, melting temperature

**Supplementary Table S2** Genotype and allele frequencies of analysed polymorphisms

| Gene | rs number | Genotype | *n* | MAF | HWE *P*-value |
|---|---|---|---|---|---|
| CYP3A4 | rs2242480 | GG | 13 | 0.147 | 0.22 |
| | | GA | 3 | | |
| | | AA | 1 | | |
| CYP3A5 | rs776746 | GG | 13 | 0.147 | 0.22 |
| | | GA | 3 | | |
| | | AA | 1 | | |
| SLCO1B1 | rs2306283 | GG | 10 | 0.235 | 0.94 |
| | | GA | 6 | | |
| | | AA | 1 | | |
| SLCO1B1 | rs4149056 | TT | 11 | 0.176 | 0.38 |
| | | TC | 6 | | |
| | | CC | 0 | | |
| SLCO1B3 | rs4149117 | GG | 8 | 0.382 | 0.12 |
| | | GT | 5 | | |
| | | TT | 4 | | |
| SLCO1B3 | rs7311358 | AA | 8 | 0.382 | 0.12 |
| | | AG | 5 | | |
| | | GG | 4 | | |
| ABCC2 | rs717620 | CC | 11 | 0.176 | 0.38 |
| | | CT | 6 | | |
| | | TT | 0 | | |
| ABCC2 | rs2273697 | GG | 15 | 0.059 | 0.80 |
| | | GA | 2 | | |
| | | AA | 0 | | |
| ABCC2 | rs3740066 | CC | 9 | 0.294 | 0.54 |
| | | CT | 6 | | |
| | | TT | 2 | | |
| UGT1A9 | rs3832043 | TT | 8 | 0.294 | 0.58 |
| | | T/- | 8 | | |
| | | -/- | 1 | | |
| UGT2B7 | rs7439366 | CC | 7 | 0.324 | 0.39 |
| | | CT | 9 | | |
| | | TT | 1 | | |

MAF, minor allele frequency; HWE, hardy-weinberg equilibrium

**Supplementary Table S3** Pharmacokinetic parameters of MPA, MPAG, and AcMPAG estimated by the non-compartmental analysis after administration of MMF alone or in combination with TAC

| **Parameter** | **MMF (mean ± SD)** | **TAC + MMF (mean ± SD)** | ***P* value** |
|---|---|---|---|
| *MPA* | | | |
| $C_{max}$ (μg/mL) | 24.5 ± 17.2 | 23.2 ± 8.3 | 0.7819 |
| $t_{max}$ (h) | 1 (1-1) | 1 (1-2) | - |
| $AUC_{0-12}$ (μg·h/mL) | 43.1 ± 23.3 | 43.1 ± 12.1 | 0.3529 |
| $AUC_{0-24}$ (μg·h/mL) | 56.1 ± 25.3 | 56.4 ± 14.2 | 0.3060 |
| $AUC_{0-48}$ (μg·h/mL) | 69.7 ± 25.7 | 69.8 ± 17.8 | 0.4586 |
| $AUC_{0-72}$ (μg·h/mL) | 73.3 ± 25.9 | 72.7 ± 18.8 | 0.5791 |
| $AUC_{0-inf}$ (μg·h/mL) | 74.7 ± 26 | 74.0 ± 19.3 | 0.5791 |
| *MPAG* | | | |
| $C_{max}$ (μg/mL) | 40.7 ± 9.9 | 41.5 ± 10.6 | 0.7743 |
| $t_{max}$ (h) | 2 (1-4) | 2 (1-4) | - |
| $AUC_{0-12}$ (μg·h/mL) | 223.7 ± 62.5 | 229 ± 56 | 0.4967 |
| $AUC_{0-24}$ (μg·h/mL) | 315.7 ± 85.7 | 329.5 ± 79.1 | 0.2085 |
| $AUC_{0-48}$ (μg·h/mL) | 404.3 ± 95.6 | 417.2 ± 95.3 | 0.3159 |
| $AUC_{0-72}$ (μg·h/mL) | 431.1 ± 97.7 | 438 ± 96.8 | 0.5905 |
| $AUC_{0-inf}$ (μg·h/mL) | 440.5 ± 98.7 | 445.1 ± 95.7 | 0.7233 |
| *AcMPAG* | | | |
| $C_{max}$ (μg/mL) | 0.7 ± 0.3 | 0.8 ± 0.3 | 0.8731 |
| $t_{max}$ (h) | 1 (1-1) | 1 (1-2) | - |
| $AUC_{0-12}$ (μg·h/mL) | 2.7 ± 1.3 | 2.8 ± 1.0 | 0.4301 |
| $AUC_{0-24}$ (μg·h/mL) | 3.6 ± 2.0 | 3.9 ± 1.5 | 0.2224 |
| $AUC_{0-48}$ (μg·h/mL) | 4.5 ± 2.8 | 4.8 ± 2.1 | 0.3654 |
| $AUC_{0-72}$ (μg·h/mL) | 4.9 ± 3.3 | 5.1 ± 2.4 | 0.6606 |
| $AUC_{0-inf}$ (μg·h/mL) | 5.5 ± 4.0 | 5.4 ± 2.6 | 0.8233 |

MMF, mycophenolate mofetil; MPA, mycophenolic acid; MPAG, MPA 7-O-glucuronide; AcMPAG, MPA acyl glucuronide; TAC, tacrolimus; SD, standard deviation; $C_{max}$, maximum concentration; $t_{max}$, time of maximum concentration; $t_{max}$ is presented as median (min-max); AUC, area under the blood concentration-time curve from time 0 to infinity or pre-specified time points; *P* value was obtained by paired *t*-test or signed rank test

**Figure legend**

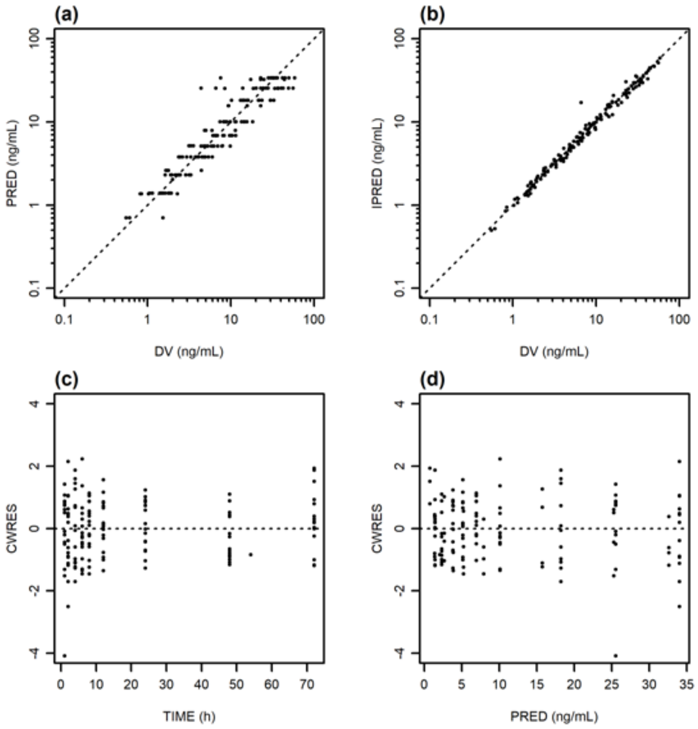


**Supplementary Figure S1** Goodness of fit plot of tacrolimus for the integrated population pharmacokinetic model. (a) population prediction (PRED) *vs.* observed concentration (DV); (b) individual prediction (IPRED) *vs.* DV; (c) conditional weighted residual (CWRES) *vs.* TIME; (d) CWRES *vs.* PRED.

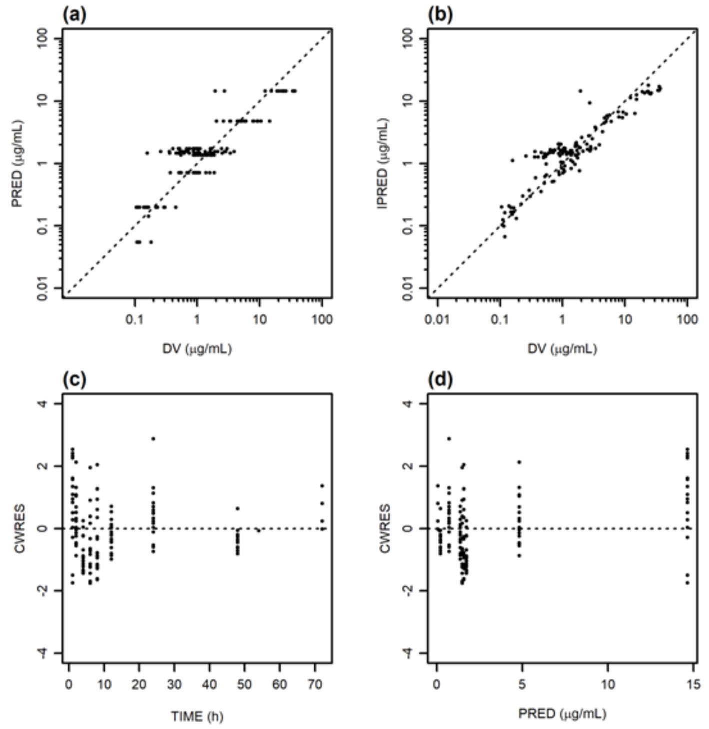


**Supplementary Figure S2** Goodness of fit plot of mycophenolic acid for the integrated population pharmacokinetic model. (a) population prediction (PRED) *vs.* observed concentration (DV); (b) individual prediction (IPRED) *vs.* DV; (c) conditional weighted residual (CWRES) *vs.* TIME; (d) CWRES *vs.* PRED.

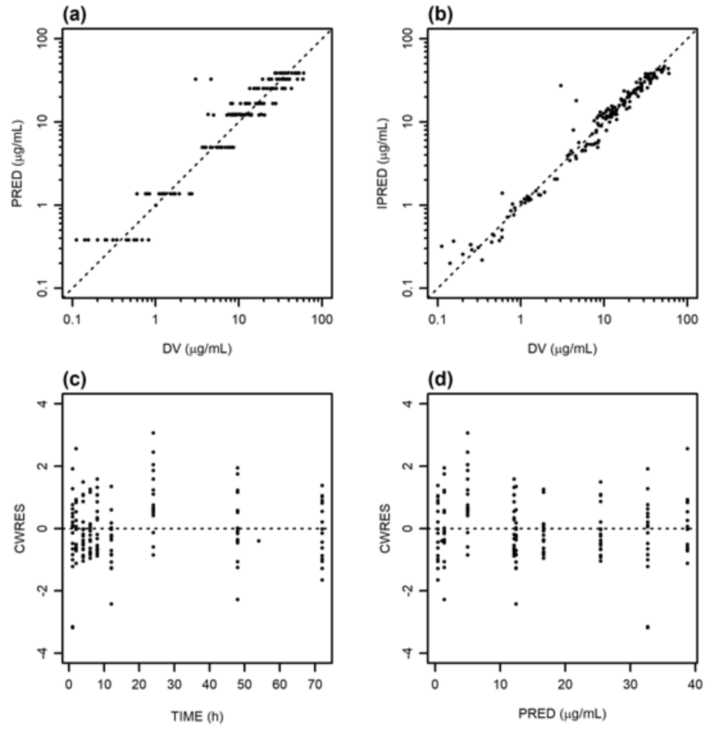


**Supplementary Figure S3** Goodness of fit plot of mycophenolic acid 7-O-glucuronide for the integrated population pharmacokinetic model. (a) population prediction (PRED) *vs.* observed concentration (DV); (b) individual prediction (IPRED) *vs.* DV; (c) conditional weighted residual (CWRES) *vs.* TIME; (d) CWRES *vs.* PRED.

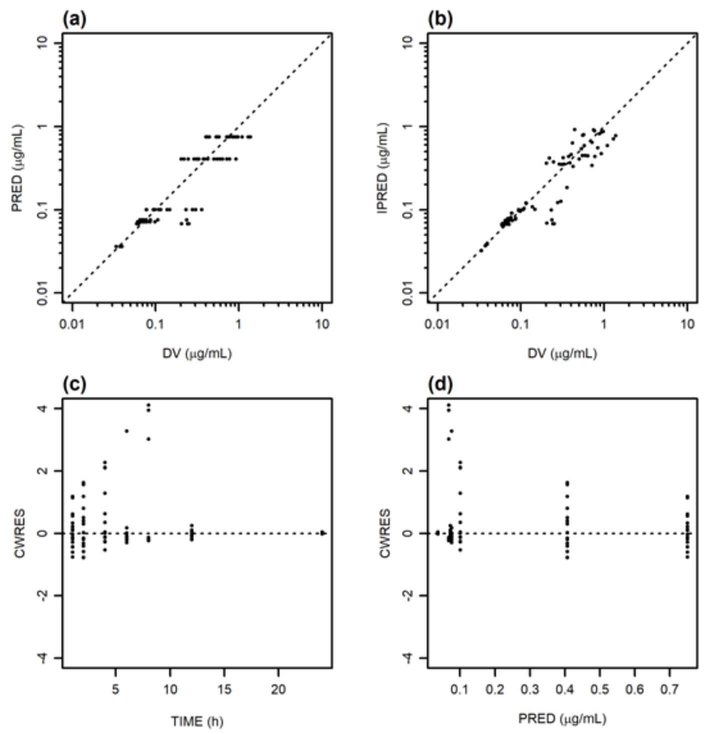


**Supplementary Figure S4** Goodness of fit plot of mycophenolic acid acyl glucuronide for the integrated population pharmacokinetic model. (a) population prediction (PRED) *vs.* observed concentration (DV); (b) individual prediction (IPRED) *vs.* DV; (c) conditional weighted residual (CWRES) *vs.* TIME; (d) CWRES *vs.* PRED.
